# P041: Trends in antiviral use in hospitalized patients following the 2009 influenza pandemic in Canada

**DOI:** 10.1186/2047-2994-2-S1-P41

**Published:** 2013-06-20

**Authors:** G Taylor, R Mitchell, A McGeer, C Frenette, K Suh, A Wong, K Katz, K Wilkinson, B Amihod, D Gravel, Canadian Nosocomial Infection Surveillance Program

**Affiliations:** 1 University of Alberta, Edmonton, Canada; 2 Public Health Agency of Canada, Ottawa, Canada; 3 University of Toronto, Toronto, Canada; 4 McGill University, Montreal, Canada; 5 University of Ottawa, Ottawa, Canada; 6 University of Saskatchewan, Saskatoon, Canada; 7 Jewish General Hospital, Montreal, Canada

## Introduction

Antiviral treatment is associated with reduced mortality, length of stay and improved clinical outcomes among patients hospitalized with influenza.

## Objectives

We wished to assess trends in use of antiviral treatment of adults hospitalized with influenza before , during and after the 2009 influenza pandemic

## Methods

CNISP has carried out prospective surveillance for laboratory confirmed influenza in hospitalized adults since 2006. These data were reviewed to assess antiviral use.

## Results

A higher proportion of adults hospitalized with laboratory-confirmed influenza received antiviral treatment during the 2009 influenza pandemic (997/1,113; 89.6%) compared with the 2010-2011 (873/1,092; 80.0%, p<0.001) and 2011-12 (391/600; 65.2%, p<0.001) influenza seasons. The decrease in antiviral us . e between the 2009 pandemic season and the 2010-11 season was statistically significant in all age groups except for inpatients >65 years. A decrease in antiviral use among inpatients >65 years was not observed until the 2011-2012 season. A median of 3 days between symptom onset and antiviral treatment was reported for all three influenza seasons. Among high-risk groups, adults with underlying medical conditions were significantly more likely to receive antiviral treatment during the pandemic than during the post-pandemic seasons (89.7% vs. 75.7%, p<0.001). A higher proportion of adults admitted to the ICU during the 2009 pandemic (94.2%) received antiviral treatment compared with the 2010-11 and 2011-12 seasons (84.6%, p<0.001). There was no difference in antiviral treatment among inpatients who died within 30 days of admission during the pandemic (84.3%) than during the post-pandemic seasons (78.9%, p=0.370).

## Conclusion

Antiviral treatment of adults hospitalized with laboratory-confirmed influenza significantly fell in the two seasons following the 2009 influenza pandemic. In order to guide strategies aimed at minimizing the impact of influenza among hospitalized adults, reasons for the decline in antiviral treatment need to be further explored.

## Disclosure of interest

None declared

